# Heat shock protein 72 (HSP72) modulates glucagon secretion via JNK inhibition in pancreatic α-cells

**DOI:** 10.1007/s13340-026-00886-6

**Published:** 2026-03-29

**Authors:** Takuro Watanabe, Tatsuya Kondo, Rintaro Yoshizumi, Nobukazu Miyakawa, Sayaka Kitano, Mary Ann Suico, Miki Sato, Masaji Sakaguchi, Motoyuki Igata, Takeshi Matsumura, Hirofumi Kai, Eiichi Araki, Naoto Kubota

**Affiliations:** 1 Department of Diabetes, Metabolism and Endocrinology, Kumamoto University Hospital, 1-1-1 Honjo, Chuo-Ward, Kumamoto, 860-8556 Japan; 2 Department of Metabolic Medicine, Faculty of Life Sciences, Kumamoto University, 1-1-1 Honjo, Chuo-Ward, Kumamoto, 860-8556 Japan; 3 Department of Diabetes, Metabolism and Endocrinology, Aso Medical Center, 1266 Kurokawa, Aso, 869-2225 Japan; 4 Department of Molecular Medicine, Faculty of Life Sciences, Kumamoto University, 5-1 Oe, Chuo-Ward, Kumamoto, 862-0973 Japan; 5 Diabetes Center, Kikuchi Medical Association Hospital, 75-3 Dairinji, Kikuchi, 861-1306 Japan; 6 Research Center for Health and Sports Sciences, Kumamoto Health Science University, 325 Izumi-Machi, Kita-Ward, Kumamoto, 861-5598 Japan; 7 Department of Medical Technology, Faculty of Health Sciences, Kumamoto Health Science University, 325 Izumi-Machi, Kita-Ward, Kumamoto, 861-5598 Japan

**Keywords:** HSP72, Glucagon, JNK, α-cell, Insulin signaling, Diabetes

## Abstract

Heat shock protein 72 (HSP72) plays a protective role against metabolic stress through suppression of c-Jun N-terminal kinase (JNK). While HSP72 has been extensively studied in insulin-sensitive tissues and pancreatic β-cells, its role in regulating glucagon secretion in α-cells remains unclear. Because glucagon secretion is enhanced by stress-responsive kinases including JNK, we hypothesized that HSP72 may negatively regulate glucagon secretion. To evaluate the effects of HSP72 on glucagon regulation, HSP72 induction was achieved using a combination of heat shock (HS) and mild electrical stimulation (MES) in *db/db* mice. In vitro, αTC cells were treated with HS + MES, transfected with HSP72 overexpression plasmids, or subjected to HSP72 knockdown. Glucagon secretion, JNK phosphorylation, insulin signaling, and expression of glucagon-related transcription factors were analyzed. Pancreatic islets were isolated from wild-type (WT) and HSP72-knockout (KO) mice to assess glucagon secretion under basal, inflammatory, and metabolic stress conditions. HS + MES-treated *db/db* mice showed reduced fasting and random glucagon levels, accompanied by increased pancreatic HSP72 expression and decreased glucagon-positive islet area. In αTC cells, HSP72 induction suppressed TNF-α–induced glucagon secretion and JNK phosphorylation while restoring insulin-induced Akt phosphorylation. HSP72 knockdown abolished these protective effects. Islets from KO mice secreted significantly more glucagon under inflammatory (TNF-α) and metabolic (high-fat diet) stress. HSP72 overexpression decreased Pax6 and MafB mRNA levels, indicating transcriptional suppression of proglucagon expression. HSP72 suppresses glucagon secretion by inhibiting JNK activation, improving insulin signaling, and downregulating key glucagon transcription factors in α-cells. These findings identify HSP72 as a novel regulator of α-cell stress responses and a potential therapeutic target for glucagon dysregulation in diabetes.

**Supplementary Information:**

The online version contains supplementary material available at 10.1007/s13340-026-00886-6.

## Introduction

As of 2021, an estimated 537 million people worldwide were living with diabetes, projected to rise to 643 million by 2030 and 783 million by 2045 [1]. Among various forms of diabetes, type 2 diabetes (T2D) accounts for the majority. T2D is a multifactorial metabolic disorder characterized by impaired insulin secretion from pancreatic β-cells and/or reduced insulin sensitivity in peripheral tissues, leading to relative insulin deficiency and chronic hyperglycemia. To fully understand the molecular mechanisms underlying T2D pathophysiology, it is important to consider intercellular communication within pancreatic islets, particularly between α- and β-cells, which are anatomically adjacent and interact through glucagon and insulin secretion at high intra-islet concentrations.

Glucagon is a 29-amino acid peptide hormone secreted by α-cells, and it plays a crucial role as a counter-regulatory hormone to insulin. It functions as an anti-hypoglycemic agent by stimulating hepatic gluconeogenesis and glycogenolysis. Recent studies have proposed that insulin and glucagon do not merely function in opposition but instead cooperate to regulate systemic energy metabolism in response to fluctuating physiological demands [2]. Glucagon secretion is typically enhanced during hypoglycemia and suppressed during hyperglycemia and is subject to regulation by both the nervous system and hormonal signals, including glucagon-like peptide-1 (GLP-1), glucose-dependent insulinotropic polypeptide (GIP), and somatostatin [2]. Additionally, insulin, zinc ions, and γ-aminobutyric acid—all secreted by β-cells—have been shown to modulate glucagon secretion, supporting the concept that β-cells regulate α-cell function in an intra-islet paracrine manner [2].

In the diabetic state, insulin resistance and sustained hyperglycemia lead to increased oxidative stress, which in turn activates stress-responsive signaling pathways such as the c-Jun N-terminal kinase (JNK) cascade. Paradoxically, this activation has been implicated in the upregulation of glucagon secretion under hyperglycemic conditions. Moreover, both insulin resistance and β-cell dysfunction have been increasingly associated with oxidative stress and endoplasmic reticulum (ER) stress [3–6], with the JNK pathway identified as a central mediator of insulin resistance [4, 7, 8]. Our research has focused on the role of the heat shock response (HSR)—particularly the induction of heat shock protein 72 (HSP72)—in the context of diabetes. We have previously demonstrated that the combination of heat shock (HS) and mild electrical stimulation (MES) effectively induces HSP72 expression, leading to suppression of JNK activation and improvements in glucose homeostasis and insulin sensitivity in both high-fat diet-induced diabetic models [9] and *db/db* mice [10]. Given that glucagon expression is positively regulated by stress signals such as JNK activation under diabetic conditions, we hypothesized that HSP72 induction suppresses glucagon expression and secretion in pancreatic α-cells. The present study was designed to elucidate this regulatory role of HSP72 and to clarify the underlying signaling mechanisms both in vivo and in vitro.

## Methods

### Cell culture and in vitro HS and MES treatment

Alpha TC 1 clone 6 cells (αTC cells) (ATCC, Manassas, VA, USA) were maintained in Dulbecco’s Modified Eagle’s Medium (DMEM) containing 25 mM glucose, 10% fetal bovine serum (FBS) and antibiotics (100 µg/mL streptomycin, 100 units/mL penicillin, 0.25 µg/mL amphotericin B) at 37℃ and 5% CO_2_. Cells were plated on 60-mm culture dishes, and at 60–80% confluency, cells were treated with HS (42℃) and MES (0.6 V/cm, 55 pulses/s, 0.1-milli second (ms) duration).

### HSP72 overexpression plasmid transfection

To construct the Myc fusion protein, pCR2.1/HSP72 containing human HSP72 (HSP70A1A) cDNA was used as a template and subcloned into pCMV-Tag5 vector. This plasmid was transfected using Lipofectamine 3000 (Invitrogen, Carlsbad, CA, USA).

### Hsp72 siRNA transfection

Hsp72 siRNA was designed; sense, *5′-GGAGCUGGAGCAGGUGUGUTT-3′* and anti-sense, *5′-ACACACCUGCUCCAGCUCCTT-3′*. GL2 (Luciferase) siRNA duplex was used as control for siRNA transfection; sense, *5′-CGUACGCGGAAUACUUCGATT-3′* and anti-sense, *5′-UCGAAGUAUUCCGCGUACGTT-3′*. Transient transfection of siRNA duplex was performed for 48 h using Lipofectamine RNAiMAX (Invitrogen, Carlsbad, CA, USA).

### Pancreatic islet isolation

Pancreatic islet isolation was performed with reference to previous protocols (4) for male wild-type and HSP72-knock out mice (global null mutation of Hspa1a/Hspa1b genes; purchased from Mutant Mouse Regional Resource Center Repository) (5).

Briefly, islets WT and HSP72KO mice were isolated by collagenase perfusion, purified by hand-picking, cultured overnight, then exposed to TNF-α or high glucose. Supernatants were collected for glucagon ELISA.

### Glucagon secretion in αTC cells

After αTC cells were transfected with control or HSP72 overexpression plasmid in DMEM containing 5.5 mM glucose, TNF-α was added to the culture medium at a concentration of 25 ng/mL and cultured for 6 h. Next, cells were incubated in HEPES-KRB, pH 7.4, containing 1% bovine serum albumin fatty acid free and 25 mM glucose for 60 min. Immediately after incubation, the supernatant was removed for assay of glucagon.

### Western blotting

For Western blotting, 40 µg of cellular protein extracts were used. Protein was resolved by SDS–polyacrylamide gel electrophoresis, and was transferred to a nitrocellulose membrane. Antibodies used: anti-phospho-JNK (Thr183/Tyr185), anti-JNK, anti-phospho-Akt (Ser473), anti-Akt, anti-Pax6 (Cell Signaling Technology, MA, USA), anti-HSP72 (StressMarq, Victoria, Canada), anti-α-tubulin clone DM1A (Millipore, MA, USA). Relative protein amounts were estimated by National Institutes of Health Image analysis software.

### RT-PCR

Total RNA was extracted from tissues using RNeasy Mini Kit (Qiagen, Hilden, Germany) and was reverse-transcribed into cDNA using Rever Tra Ace® qPCR RT Master Mix (TOYOBO, Osaka, Japan). RT-PCR was performed using Go Taq® qPCR Master Mix (Promega, Madison, USA), 18 s mRNA was used as a normalization control for mRNA transcriptional levels. Primer sequences used for RT-PCR are specific primers for 18 s upstream primer (*5′-CGATCCGAGGGCCTCACTA-3′*) and downstream primer (*5′-AGTCCCTGCCCTTTGTACACA-3′*), HSP72 upstream primer (*5′-TGGTGCTGACGAAGATGAAG-3′*) and downstream primer (*5′-AGGTCGAAGATGAGCACGTT-3′*), Pax6 upstream primer (5*′-CCAACGACAATATACCCAGTGTGTC-3′*) and downstream primer (*5′-TGTTGCTGGCAGCCGTCTTGCGTG-3′*), MafB upstream primer (*5′-TGAATTTGCTGGCACTGCTG-3′*) and downstream primer (*5′-AAGCACCATGCGGTTCATACA-3′*). Proglucagon upstream primer (*5′*-*AGGCGTCAGGCGTCATCTG-3′*) and downstream primer (*5′-GGTTCCTCTTGGTGTTCATCA-3′*).

### Immunofluorescent staining in pancreatic islet

Pancreas isolated from mice were fixed in 10% natural buffered formalin and placed in frozen blocks using OCT compound (Sakura Fineteck Japan. Tokyo). Pancreas Sects. (7 µm) from frozen blocks were immunohistochemically stained following standard procedures using primary antibodies for Glucagon (proteintech®, Illinois, USA), HSP72 (StressMarq, Victoria, Canada), Insulin (SANTA CRUZ BIOTECHNOLOGY, Dallas, USA). We determined glucagon-positive areas and insulin-positive areas using a BZ Ⅱ All-in-One Fluorescence Microscope (Keyence, Osaka, Japan).

### ELISA assays

Glucagon and insulin concentration levels were assayed with ELISA kits (Insulin/Glucagon, FUJUFILM Wako Pure Chemical Corporation, Osaka, Japan.) according to the instruction manuals.

### Animals and in vivo intervention

Male *db/db* mice were treated with HS (42 °C) plus MES (0.6 V/cm, 55 pulses/s, 0.1 ms) three times weekly for 11 weeks starting at 6 weeks. Sham-treated mice served as controls. Intraperitoneal insulin (1 U/kg) and glucose (2 g/kg) tolerance tests were performed at 13 and 14 weeks. Blood glucose was measured enzymatically up to 600 mg/dL; higher values were measured after 1:1 saline dilution. At 17 weeks, sera and pancreata were collected. Procedures were approved by the Kumamoto University Animal Experimentation Committee (A2023-012).

### Statistical analysis

All data are presented as means ± standard deviation (SD). Statistical significance was determined by unpaired two-tailed Student’s *t*-test for two-group comparisons, or by one-way ANOVA followed by Bonferroni’s post hoc test for multiple-group comparisons. Values of P less than 0.05 were considered statistically significant.

## Results

### The effect of HS + MES on glucagon secretion and expression in db/db mice

In our previous study, we reported that HS combined with MES induced HSP72 expression in pancreatic β-cells of *db/db* mice, resulting in improved β-cell mass and function. [10].

In the present study, we aimed to investigate the effect of HS + MES on glucagon secretion in *db/db* mice. Male *db/db* mice were subjected to HS + MES three times a week from 6 to 17 weeks of age. The mice were fed a normal chow diet (NCD) and had ad libitum access to water. Serum glucagon levels were measured, and the animals were euthanized at 17 weeks of age for dissection, followed by immunostaining of pancreatic islets.

**Fig. 1:**
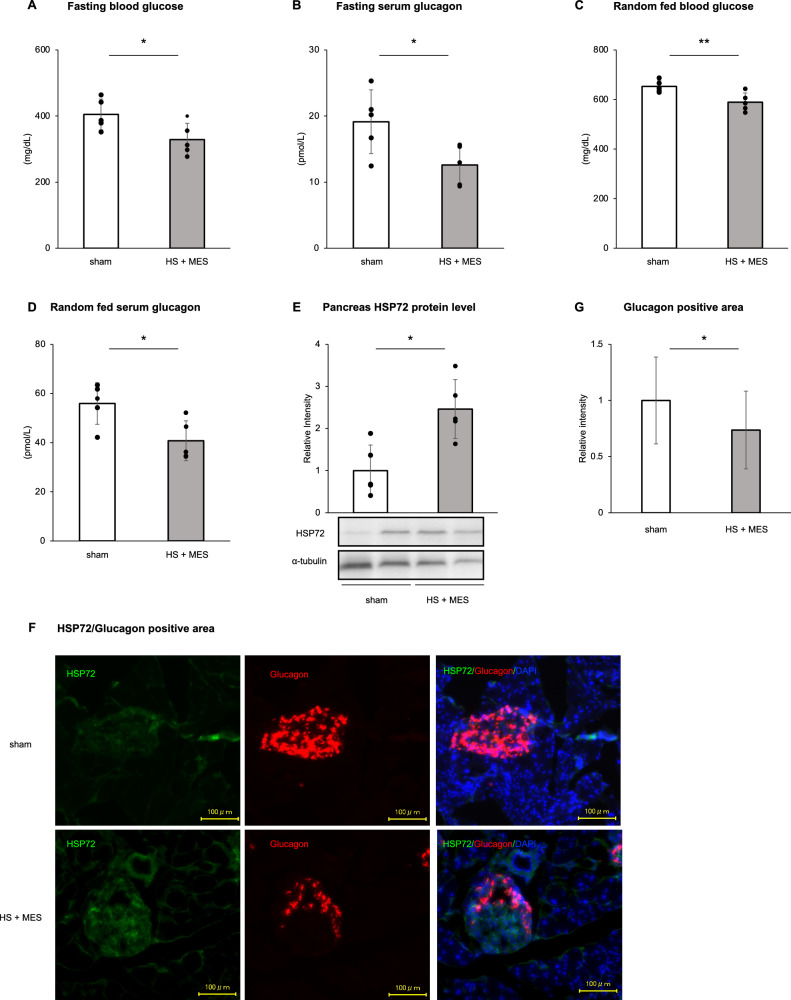
Fasting blood glucose (**A**), fasting serum glucagon levels (**B**), random-fed blood glucose (**C**), and random-fed serum glucagon levels (**D**) were measured in *db/db* mice fed a normal chow diet (NCD) and subjected to either sham treatment or combined heat shock (HS) and mild electrical stimulation (MES) (n = 5 per group). Pancreatic tissues were collected, and HSP72 protein expression was assessed by Western blotting (**E**). Pancreatic islets were immune-stained for glucagon and HSP72 (**F**), and the glucagon-positive area was quantified (**G**). Data are expressed as means ± standard deviation (SD). Statistical significance was determined using an unpaired t-test: *P < 0.05, **P < 0.01

The results showed that fasting blood glucose levels were significantly lower in the HS + MES group compared to the sham-treated group (404 ± 46 mg/dL vs. 328 ± 49 mg/dL, p = 0.03; Fig. 1A). Similarly, fasting serum glucagon levels were significantly reduced in the HS + MES group (19.1 ± 4.8 pmol/L vs. 12.6 ± 3.0 pmol/L, p = 0.03; Fig. 1B). Furthermore, random-fed blood glucose and serum glucagon levels were also significantly lower in the HS + MES group compared to the sham group (random-fed blood glucose: 652 ± 23 mg/dL vs. 589 ± 37 mg/dL, p = 0.01; Fig. 1C; random-fed serum glucagon: 55.9 ± 8.5 pmol/L vs. 40.8 ± 8.1 pmol/L, p = 0.02; Fig. 1D). There were no significant differences in body weight or food intake between the two groups, and blood glucose and glucagon levels were not affected by these factors, as confirmed by supplementary analysis (Supplementary Fig. 1A,B).

The protein expression levels of HSP72 in the whole pancreas homogenate were significantly 2.4-fold higher in the HS + MES group compared to the sham-treated group (Fig. 1E). Immuno-staining for glucagon in pancreatic islets revealed that the glucagon-positive area was 26 ± 4.1% lower in the HS + MES group compared to the sham group (p = 0.01; Fig. 1F, G). Immunofluorescence analysis further demonstrated upregulation of HSP72 within islets following HS + MES treatment, accompanied by a reduction in glucagon-positive area (Fig. 1F). Additionally, fasting and random-fed serum insulin levels were elevated in the HS + MES group compared to the sham group (Supplementary Fig. 1C, D). The observed reduction in serum glucagon secretion could be due to the paracrine action of insulin in addition to the induction of HSP72 expression. Therefore, the results suggest that HS + MES treatment attenuates glucagon secretion in both the fasting and random-fed states in *db/db* mice. This effect appears to be mediated by HSP72 induction and the subsequent decrease in glucagon expression in the pancreatic islets.

### Glucagon secretion in αTC cells

**Fig. 2:**
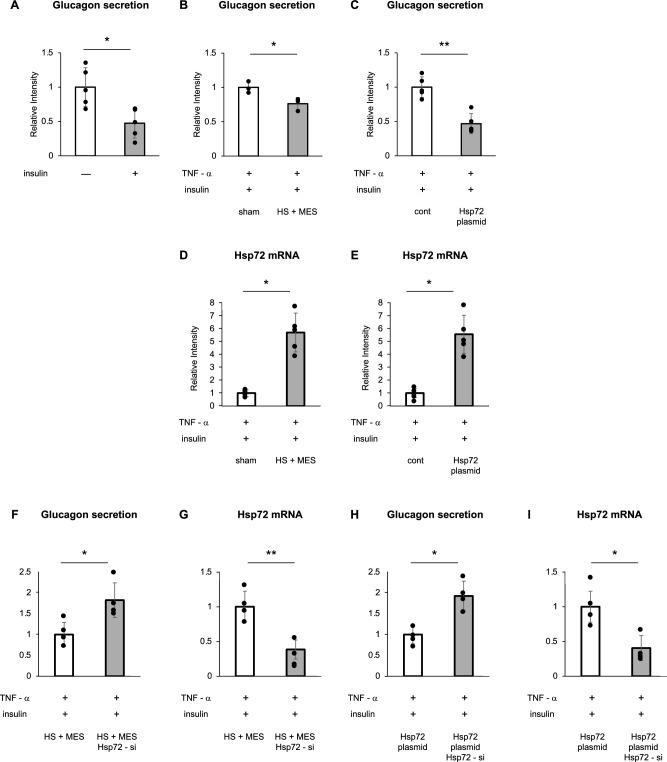
**A** αTC cells were incubated in HEPES-KRB buffer containing 25 mM glucose with or without 100 nM insulin for 1 h, and glucagon secretion into the supernatant was measured (n = 5 per group). **B** αTC cells were subjected to heat shock (HS) and mild electrical stimulation (MES) for 120 min to induce HSP72 expression, followed by stimulation with 25 ng/mL TNF-α for 6 h. Cells were then incubated in HEPES-KRB buffer containing 25 mM glucose and 100 nM insulin for 1 h, and glucagon secretion was measured (n = 3 per group). **C** αTC cells were transfected with either a control plasmid or an Hsp72 overexpression plasmid and stimulated with 25 ng/mL TNF-α for 6 h. Glucagon secretion was subsequently assessed after 1-h incubation in HEPES-KRB buffer containing 25 mM glucose and 100 nM insulin (n = 5 per group). **D**, **E** Hsp72 mRNA expression levels following HS + MES treatment were quantified by real-time PCR. **F** Effects of HS + MES and Hsp72 siRNA on TNF-α–induced glucagon secretion. **G** Hsp72 mRNA expression levels following plasmid transfection. **H** Effects of Hsp72 overexpression and Hsp72 siRNA on TNF-α–induced glucagon secretion. **I** Confirmation of Hsp72 knockdown efficiency by real-time PCR. Data are presented as means ± SD. Statistical significance was determined using unpaired t-test or one-way ANOVA followed by appropriate post hoc testing, as indicated in the figure. *P < 0.05; **P < 0.01

An inverse association between HSP72 expression and glucagon secretion was observed in vivo. To further investigate the role of HSP72 in pancreatic α-cells, glucagon secretion was evaluated in vitro using αTC cells, a murine α-cell line. Initially, we examined the association between JNK activation and glucagon secretion, focusing on the potential involvement of insulin signaling. To examine this, αTC cells were cultured in DMEM containing 5.5 mM glucose, then supplemented with 25 mM glucose for 1 h, in the presence or absence of 100 nM insulin. Glucagon levels in the culture supernatant were subsequently measured. The results demonstrated that glucagon secretion was significantly reduced in the presence of insulin, indicating an inhibitory effect of insulin on glucagon release (Fig. 2A).

Next, to assess the role of HSP72 in glucagon secretion, HSP72 expression was induced in αTC cells via HS + MES treatment or transfection with an HSP72 overexpression plasmid. Following HS + MES or sham treatment, cells were stimulated with 25 ng/mL tumor necrosis factor-α (TNF-α) for 6 h in the presence of 100 nM insulin. Glucagon secretion was significantly lower in the HS + MES-treated group compared to the sham-treated control (Fig. 2B).

In a parallel experiment, αTC cells were transfected with either a control plasmid or an HSP72 overexpression plasmid. After transfection, cells were exposed to 25 ng/mL TNF-α for 6 h in the presence of 100 nM insulin. Consistent with the previous findings, glucagon secretion was significantly reduced in the HSP72-overexpressing group relative to the control plasmid group (Fig. 2C).

Hsp72 mRNA expression was significantly increased by HS + MES treatment or Hsp72 plasmid transfection (Fig. 2D, E). Under TNF-α stimulation, glucagon secretion was significantly suppressed by HS + MES or Hsp72 overexpression; however, siRNA-mediated knockdown of Hsp72 effectively reduced Hsp72 mRNA levels (Fig. 2G, I) and attenuated the suppressive effect on glucagon secretion (Fig. 2F, H).

These results indicate that insulin suppresses glucagon secretion under high-glucose conditions. Furthermore, HSP72 induction—either by HS + MES or by overexpression—significantly reduced glucagon secretion in αTC cells (Fig. 2B, C).

Taken together, these results demonstrate that HSP72 induction suppresses glucagon secretion in αTC cells, supporting a direct role for HSP72 in the regulation of glucagon secretion at the cellular level.

### Inhibitory effect of HSP72 on JNK phosphorylation in αTC cells

Previous findings demonstrated that increased expression of HSP72 suppresses glucagon secretion. To elucidate the underlying mechanism, we focused on the phosphorylation of JNK, a known target of HSP72. HSP72 has been reported to inhibit JNK phosphorylation, and prior studies have shown that hyperglycemia enhances JNK activity in pancreatic α-cells, impairing insulin signaling and promoting glucagon secretion [2]. Therefore, we investigated whether HSP72 expression regulates JNK activation in pancreatic α-cells in vitro***.***

**Fig. 3:**
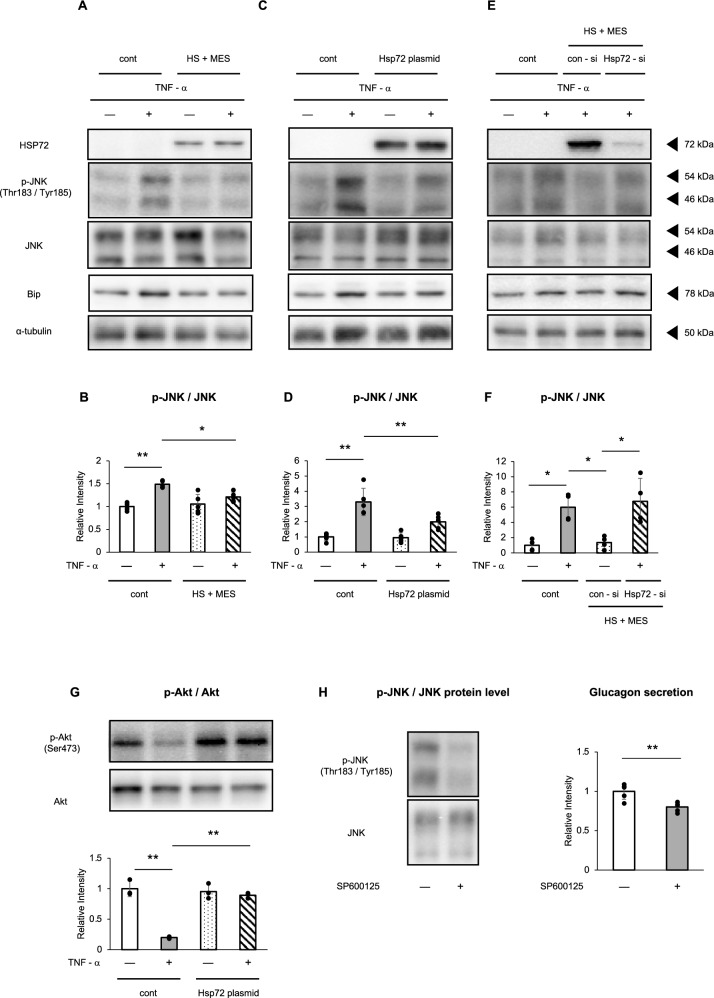
αTC cells were treated with heat shock (HS) and mild electrical stimulation (MES) for 120 min to induce HSP72 expression, followed by stimulation with 25 ng/mL TNF-α for 6 h or left untreated. Protein expression levels of HSP72, phosphorylated JNK (p-JNK), total JNK, and BiP were assessed by Western blotting (**A**). Quantification of JNK phosphorylation from (A) (**B**. n = 5 per group). αTC cells were transfected with either a control plasmid or an HSP72 overexpression plasmid, followed by treatment with 25 ng/mL TNF-α for 6 h or without, and protein expression levels of HSP72, p-JNK, JNK, and BiP were analyzed by Western blotting (**C**). Quantification of JNK phosphorylation from (C) (**D**. n = 4 per group). After transfection with control siRNA or HSP72-specific siRNA, αTC cells were treated with HS and MES to modulate HSP72 expression, and stimulated with 25 ng/mL TNF-α for 6 h or left untreated. Western blotting was used to assess the expression of HSP72, p-JNK, JNK, and BiP (**E**). Quantification of JNK phosphorylation from (**F**. n = 4 per group). Phosphorylation of Akt was evaluated following stimulation with 100 nM insulin for 10 min under the same experimental conditions (**G**. n = 3 per group). αTC cells were pretreated with 20 μM SP600125, a JNK inhibitor, for 1 h, followed by TNF-α stimulation. JNK phosphorylation and glucagon secretion in the supernatant were measured (**H**. n = 4 per group). Data are presented as means ± standard deviation (SD). Statistical significance was determined by one-way ANOVA followed by Bonferroni’s post hoc test: *P < 0.05, **P < 0.01

We have previously reported that the combination of HS and MES inhibits JNK phosphorylation via induction of HSP72 in MIN6 β-cells [10]. To examine whether a similar mechanism operates in α-cells, αTC cells were treated with HS + MES to induce HSP72 expression, followed by stimulation with 25 ng/mL TNF-α. This treatment suppressed TNF-α–induced JNK phosphorylation, as confirmed by immunoblotting (Fig. 3A, B). HSP72 also reduced BiP expression, suggesting potential modulation of ER stress pathways (Fig. 3A, C and E).

Next, to confirm the role of HSP72 in modulating JNK activation, αTC cells were transfected with either a control or HSP72-overexpressing plasmid and then treated with TNF-α (25 ng/mL, 6 h). TNF-α markedly increased JNK phosphorylation, whereas HSP72 overexpression significantly attenuated this effect (Fig. 3C and D).

To further validate these findings, HSP72 expression was knocked down using HSP72-specific siRNA. siRNA transfection reduced HSP72 protein levels by approximately 90%, which abolished the inhibitory effect of HSP72 on TNF-α–induced JNK phosphorylation (Fig. 3E and F). Additionally, we evaluated the expression of binding immunoglobulin protein (BiP), a marker of ER stress, and found that its expression was also regulated by HSP72 in a manner similar to JNK.

To assess downstream insulin signaling, we evaluated Akt phosphorylation. In cells overexpressing HSP72 (as described in Fig. 3C and D), insulin (100 nM) was administered for 10 min, and Akt phosphorylation was measured. TNF-α treatment inhibited Akt phosphorylation, but this inhibition was reversed in HSP72-overexpressing cells (Fig. 3G), indicating restoration of insulin signaling.

To determine whether the observed effects on glucagon secretion are mediated through JNK, we utilized SP600125, a broad-spectrum JNK inhibitor. αTC cells were pretreated with SP600125 (20 μM) for 1 h, followed by TNF-α stimulation. SP600125 reduced JNK phosphorylation by approximately 60%, and this reduction was accompanied by a significant decrease in glucagon secretion (Fig. 3H).

Collectively, these results demonstrate that HSP72 protects pancreatic α-cells by suppressing JNK activation and restoring insulin signaling pathways, and ultimately contributes to the inhibition of glucagon secretion in vitro.

### Glucagon secretion in pancreatic islets

**Fig. 4:**
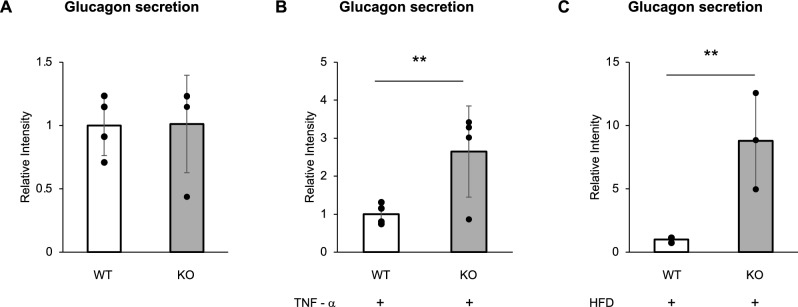
Glucagon secretion from isolated pancreatic islets of C57BL/6 J wild-type (WT) and HSP72 knockout (KO) mice (n = 4 per group) fed a normal chow diet (NCD) was measured after incubation in HEPES-KRB buffer containing 25 mM glucose without TNF-α stimulation (**A**). Glucagon secretion was similarly assessed following exposure to 25 ng/mL TNF-α for 6 h under the same glucose conditions (**B**). Glucagon secretion from isolated pancreatic islets of WT and KO mice (n = 3 per group) fed a high-fat diet (HFD) for 22 weeks was evaluated in 25 mM glucose without TNF-α (**C**). Data are presented as means ± standard deviation (SD). Statistical analysis was performed using an unpaired t-test. **P < 0.01

To investigate glucagon secretion from pancreatic α-cells under physiological conditions, 24-week-old male C57BL/6 J wild-type (WT) and HSP72 knockout (HSP72KO; KO) mice, both maintained on a NCD, were utilized. Pancreatic islets were isolated from these animals and subjected to in vitro analysis. The isolated islets were cultured overnight in RPMI medium containing 5.5 mM glucose and subsequently transferred to HEPES-buffered KRB solution containing 25 mM glucose. After 1 h of incubation, the culture supernatant was collected, and glucagon concentrations were measured. Under these basal conditions, there was no significant difference in glucagon secretion between WT and KO islets (Fig. 4A).

To assess the effect of inflammatory stress on α-cell glucagon secretion, isolated pancreatic islets from WT and KO mice were exposed to 25 ng/mL TNF-α for 6 h. Glucagon levels in the supernatant were significantly higher in the KO group compared to the WT group (Fig. 4B), indicating that HSP72 deficiency enhances glucagon secretion in response to pro-inflammatory stimuli.

To further examine glucagon secretion under metabolic stress conditions in vivo, 8-week-old male WT and KO mice were fed a high-fat diet (HFD) for 22 weeks. At 30 weeks of age, pancreatic islets were isolated from these mice, and glucagon secretion was measured using the same protocol as above, in the absence of TNF-α. Islets from KO mice secreted significantly higher levels of glucagon compared to those from WT mice (Fig. 4C), suggesting that HSP72 mitigates the effects of long-term metabolic stress on α-cell glucagon secretion.

Together, these results indicate that while HSP72 does not significantly affect glucagon secretion under basal conditions, it plays a critical role in suppressing glucagon hypersecretion in response to both inflammatory and metabolic stress.

### HSP72 and glucagon transcription factor

To explore the relationship between HSP72 expression and transcriptional regulation of glucagon in pancreatic α-cells, we examined the expression of key glucagon transcription factors following HSP72 overexpression. αTC cells were cultured in DMEM containing 5.5 mM glucose and subsequently transfected with either a control plasmid or an HSP72 overexpression plasmid. After transfection, the cells were treated with 25 ng/mL TNF-α for 6 h, followed by incubation in HEPES-buffered KRB solution containing 25 mM glucose and 100 nM insulin for 1 h. Total RNA was then extracted from the cells for mRNA expression analysis.

**Fig. 5:**
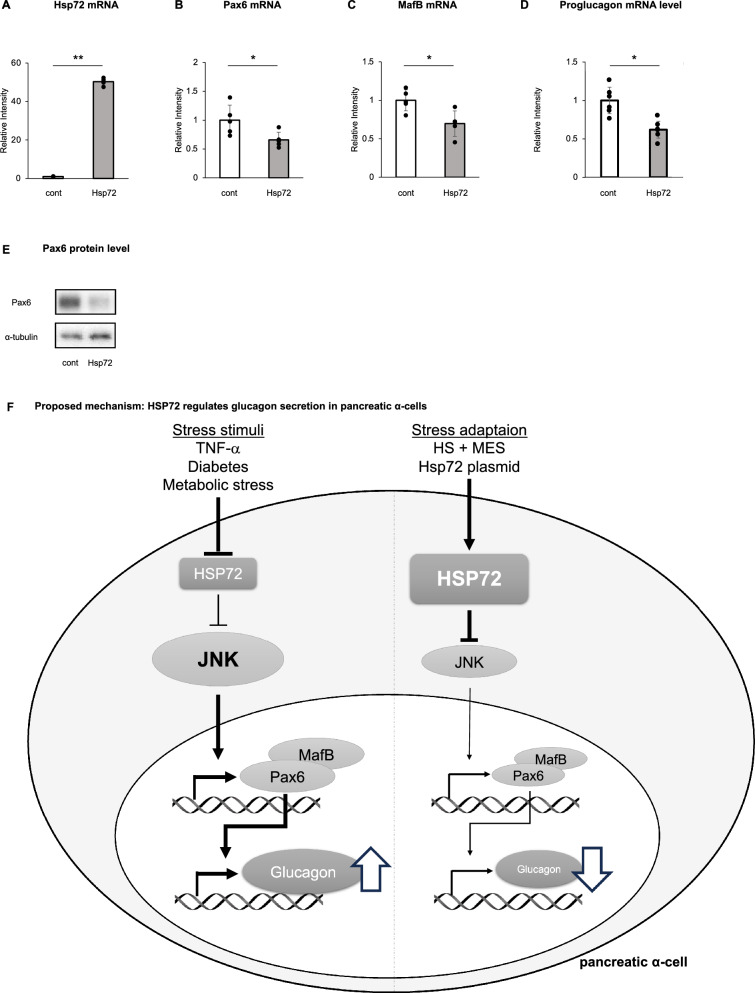
The mRNA levels of HSP72 (**A**), Pax6 (**B**), MafB (**C**), and proglucagon (**D**), as well as protein levels of Pax6 (**E**) were measured in αTC cells following stimulation with 25 ng/mL TNF-α for 6 h, followed by incubation in HEPES-KRB buffer containing 25 mM glucose and 100 nM insulin for 1 h. Data are presented as means ± standard deviation (SD). Statistical significance was assessed using an unpaired t-test. *P < 0.05, **P < 0.01. Schematic illustration (**F**) summarizing the proposed signaling pathway identified in this study. Under metabolic and inflammatory stress conditions (e.g., TNF-α stimulation and diabetic milieu), c-Jun N-terminal kinase (JNK) signaling is activated in pancreatic α-cells, leading to enhanced expression of glucagon-related transcription factors, including Pax6 and MafB, and increased glucagon production and secretion. Induction of HSP72, achieved by heat shock plus mild electrical stimulation (HS + MES) or HSP72 overexpression, suppresses JNK activation. Attenuation of JNK signaling reduces Pax6 and MafB expression, resulting in decreased proglucagon transcription and subsequent suppression of glucagon secretion. This model integrates the in vivo and in vitro findings and highlights HSP72 as a stress-responsive modulator of α-cell glucagon regulation

Quantitative PCR revealed that HSP72 mRNA expression was elevated approximately 50-fold in the HSP72 overexpression group compared to controls (Fig. 5A). Notably, the mRNA levels of glucagon-associated transcription factors Pax6 and MafB were significantly downregulated in the HSP72 overexpression group (Fig. 5B and C). In parallel with that, proglucagon mRNA levels were significantly reduced in HSP72-overexpressing cells (Fig. 5D), indicating that HSP72 suppresses glucagon synthesis at the transcriptional level. In addition, Western blot analysis confirmed that Pax6 protein levels were similarly reduced under the same experimental conditions (Fig. 5E).

These findings suggest that the inhibitory effect of HSP72 on glucagon secretion may, at least in part, be mediated through the suppression of transcription factors essential for glucagon gene expression.

## Discussion

Our study demonstrates that HSP72 induction suppresses glucagon expression and secretion in pancreatic α-cells by inhibiting JNK activation and enhancing insulin signaling. To date, no studies have reported a direct regulatory relationship between HSP72 and glucagon expression or secretion. However, given that glucagon production is known to be influenced by cellular stress signals—including activation of JNK pathway—we hypothesized that HSP72 may play a role in this regulatory mechanism.

In the present study, we demonstrated that the induction of HSP72 by combined HS and MES resulted in decreased glucagon expression and secretion in pancreatic islets of diabetic mice. Furthermore, in αTC cells, HSP72 overexpression suppressed TNF-α–induced JNK phosphorylation and enhanced insulin signaling.

These findings suggest that HSP72 induction suppresses stress-induced JNK activation, leading to downregulation of Pax6 and MafB, reduction of proglucagon transcription, and consequent attenuation of glucagon secretion. This supports a growing body of evidence implicating inflammatory pathways—particularly those involving TNF-α, JNK activation, and insulin resistance—in the pathophysiology of diabetes [11]. Previous studies have demonstrated that HSP72 suppresses JNK activation in multiple insulin-sensitive tissues, such as skeletal muscle, adipose tissue, and pancreatic β-cells, thereby contributing to improved insulin signaling and glucose homeostasis [10]. Our results demonstrate that HSP72 attenuates glucagon secretion in αTC cells. Overexpression of HSP72 enhances insulin-induced suppression of glucagon secretion, a process that appears to involve HSP72-mediated dephosphorylation of JNK.

In fact, HSP72 inhibits JNK activation through multiple mechanisms, such as direct inhibition [12], blocks upstream stress-activated protein kinase/extracellular signal-regulated kinase kinase 1 [12], suppression of mitogen-activated protein kinase kinase 1/7 [13], activation of mitogen-activated protein kinase phosphatase 1/3 [14] and suppression of dual leucine zipper-bearing kinase-1 [15].

Moreover, the suppression of glucagon expression and secretion by HSP72 may involve the downregulation of glucagon transcription factors, such as Pax6 and MafB mRNA.

The paired and homeodomain-containing transcription factor Pax6 has been shown to play critical roles in pancreatic development, β-cell function, cell survival, as well as insulin biosynthesis and secretion. Mice lacking Pax6 exhibit a significant reduction in islet cell numbers, suggesting that Pax6 is essential for the differentiation of multiple endocrine cell types within the islets [16, 17]. Pax6 protein functions as a transcription factor that binds to a conserved element present in the promoters of glucagon, insulin, and somatostatin genes, and has been shown to transactivate both glucagon and insulin promoters [16]. While Pax6 is crucial for pancreas development, insulin biosynthesis, and glucose-induced insulin secretion (GSIS), it is downregulated in islets from individuals with type 2 diabetes [18, 19]. On the other hand, high-glucose increases Pax6 expression through JNK pathway in β-cells [20].

In our α-cell model, induction of HSP72 reduces Pax6 mRNA expression, which may be upregulated by JNK activation in response to stress signals. Although the transcriptional regulation of Pax6 in α-cells remains unclear, the downregulation of Pax6 transcription following HSP72 induction is associated with decreased glucagon expression and secretion. The precise mechanism underlying this transcriptional regulation should be further investigated in future studies.

MafB is a basic leucine zipper transcription factor that plays crucial roles in development and differentiation processes. While MafB protein is present throughout the lifespan of α-cells in the islets, it is only expressed in murine β-cells during embryonic development, except for transient expression during pregnancy, which facilitates β-cell expansion [21, 22]. MafB expression is downregulated at the transcriptional level through the JNK and p38 MAP kinase pathways in osteoclasts [23]. Furthermore, JNK has been shown to promote MafB degradation in COS7 cells, suggesting that JNK may negatively regulate MafB at both the mRNA and protein levels [23]. Alterations in α-cell identity genes, such as ARX and MafB, were observed in type 1 diabetes, with upregulation of cytokine-regulated genes and genes involved in glucagon biosynthesis and processing [24]. In an α-cell-specific MafB conditional knockout mouse model, both α-cell number and glucagon production were significantly reduced [25]. In endocrine cell-specific, tamoxifen-inducible MafB knockout mice, both insulin⁺ and glucagon⁺ cells decreased at birth, but only insulin⁺ cells recovered by adulthood, indicating that MafB is required for sustained glucagon production in α-cells [26]. In our α-cell model, HSP72 induction decreased MafB mRNA, likely by counteracting stress-induced JNK activation; however, the mechanisms of MafB regulation in α-cells remain to be clarified.

Several limitations should be acknowledged. First, direct promoter binding of Pax6 or MafB to the proglucagon gene was not examined. Second, the use of a single α-cell line may not fully reflect primary human α-cell physiology. Third, although HS + MES induces HSP72, potential off-target stress responses cannot be completely excluded.

In summary, we demonstrated that diabetic stress conditions, modeled by HFD feeding in vivo or TNF-α stimulation in vitro, increase glucagon expression and secretion via JNK activation. These effects were exacerbated in the absence of HSP72. Conversely, HSP72 induction suppressed JNK activation, leading to reduced Pax6 and MafB expression, which may contribute to transcriptional downregulation of proglucagon and consequent reduction in glucagon secretion in αTC cells. Collectively, these findings extend the known protective roles of HSP72 beyond β-cells and support the concept that targeting the HSP72–JNK axis may represent a novel therapeutic strategy for glucagon dysregulation in diabetes.

## Supplementary Information

Below is the link to the electronic supplementary material.

Supplementary material

## Data Availability

The data sets generated during and/or analyzed during the current study are available from the corresponding author upon reasonable request.

## Declarations

### Approval date of registry and the registration no. of the study/trial

N/A.

### Animal Studies

Yes.
