# Supplementary figures and images for: Heat shock protein 72 (HSP72) modulates glucagon secretion via JNK inhibition in pancreatic α-cells
Source: Diabetol Int. 2026 Mar 29;17(2):34. doi: 10.1007/s13340-026-00886-6 (PMC13033473; doi:10.1007/s13340-026-00886-6)

Supplementary Figure.1

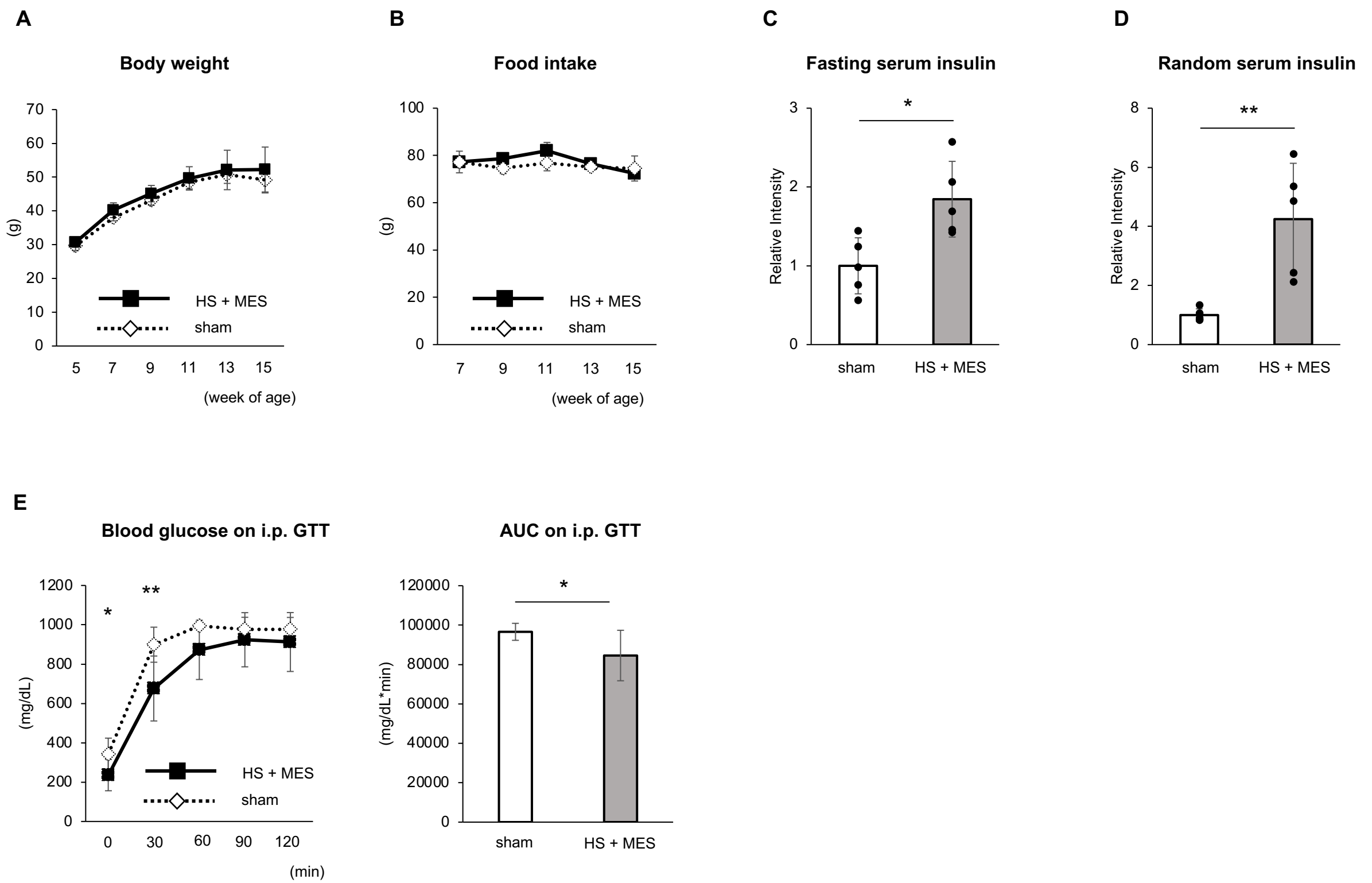

Supplement: Supplementary file 1 — Supplementary material [file 13340_2026_886_MOESM1_ESM.pdf]
